# Temporal profiles of cortisol accumulation and clearance support scale cortisol content as an indicator of chronic stress in fish

**DOI:** 10.1093/conphys/coz052

**Published:** 2019-10-11

**Authors:** Frédéric Laberge, Irene Yin-Liao, Nicholas J Bernier

**Affiliations:** Department of Integrative Biology, University of Guelph, 50 Stone Road East, Guelph, ON, Canada, N1G 2WI

**Keywords:** cortisol, goldfish (*Carassius auratus*), implant, integrated measure of stress, scales, unpredictable chronic stress

## Abstract

The development of chronic stress indicators for fish is of great interest, but appropriate non-invasive methods are lagging those used in terrestrial vertebrates. Here, we explore the possibility that levels of the stress hormone cortisol in scales could be used as a chronic stress indicator. Three experiments were conducted to assess the temporal profiles of cortisol rise and fall in plasma and scales of goldfish (*Carassius auratus*) in response to stressors of varying intensity and duration. Results show that a single acute air emersion stressor does not influence scale cortisol content. In contrast, relative to plasma levels, the fall in scale cortisol content following a high-dose cortisol implant is delayed by at least 8 days, and the rise and fall in scale cortisol content in response to unpredictable chronic stress are delayed by at least 7 days. Also, scale cortisol content is spatially heterogeneous across the body surface of goldfish. Overall, since high and sustained circulating cortisol levels are needed to influence scale cortisol content and the rates of cortisol accumulation and clearance are much slower in scales than in plasma, our results show that scales can provide an integrated measure of cortisol production and serve as a chronic stress indicator.

## Introduction

Glucocorticoids are recognized as key stress hormones across vertebrates. Released into the circulation following activation of the neuroendocrine stress axis in response to actual or perceived threats, glucocorticoids have a broad number of physiological effects on multiple target tissues (Sapolsky *et al.*, 2000; Denver, 2009). Since these glucocorticoid-mediated effects help vertebrates counter the impact of stressors and maintain their homeostatic state, they are generally considered to be adaptive. However, it is also well known that sustained levels of glucocorticoids can have deleterious effects (Schreck, 2000; McEwen and Wingfield, 2003). Therefore, determining the relationship between glucocorticoids and animal condition is of great interest, and levels of glucocorticoid hormones in wildlife have been used as physiological indicators of environmental change and anthropogenic disturbances (Pankhurst, 2011; Baker *et al.*, 2013; Wingfield, 2013; Romero and Wingfield, 2016).

Although plasma samples have traditionally been used to quantify glucocorticoid levels and monitor the response to stressors, the capacity of this measure to assess non-invasively the cumulative activity of the neuroendocrine stress axis is limited (Cook, 2012). As a result, considerable effort has recently gone into the development of minimally invasive methods that provide an integrative measure of stress hormone levels. These methods have exploited the propensity of steroid glucocorticoid hormones to accumulate in inert or relatively inert biological materials like hair (Bryan *et al.*, 2013a, b; Staufenbiel *et al.*, 2013; Wester and van Rossum, 2015), feathers (Koren *et al.*, 2012; Berk *et al.*, 2016), earplug (Trumble *et al.*, 2013), baleen (Hunt *et al.*, 2014), blubber (Trana *et al.*, 2015) or waste materials in faecal matter (Harper and Austad, 2000; Palme *et al.*, 2005). The high stability of steroid molecules allows for robust measurements even after time delays between shedding or collection and analysis (Kalbitzer and Heistermann, 2013; Nalla *et al.*, 2015).

These methods evidently show a lot of promise, but they unfortunately leave out or are difficult to use with fish, the most diverse group of vertebrates. Cortisol is the primary glucocorticoid in fish (Bernier *et al.*, 2009), and the study of stress in fish has benefited from the development of non-invasive measurement of cortisol levels in water (Scott and Ellis, 2007), but this method is only applicable under controlled conditions. Analysis of hormone content in mucus is a potential avenue (Bertotto *et al.*, 2010; Guardiola *et al.*, 2016; De Mercado *et al.*, 2018; Fernández-Alacid *et al.*, 2018; Khansari *et al.*, 2018); however, the significant correlation found between plasma and mucus cortisol levels in response to short-term disturbances suggests that mucus cortisol levels may have limited usefulness as an indicator of chronic stress. In contrast, fish scales show promise as an integrative measure of stress hormone levels based on the work of Aerts *et al.* (2015) who showed that cortisol accumulates in the scales of cortisol-fed and stressed common carp. The presence of cortisol in scales was also recently confirmed in goldfish by Carbajal *et al.* (2018) and in milkfish (*Chanos chanos*) by Hanke *et al.* (2019). Further, Carbajal *et al.* (2019) showed recently that scale cortisol content can vary across seasons in wild Catalan chub (*Squalius laietanus*).

Fish scales come in various forms, with a phylogenetic trend for gradual loss of heavy layers of dentine, vascular bone and enamel during the evolution of bony fishes (Kardong, 2009). The scales of derived teleost fishes have only retained layered lamellar bone from ancestors. These acellular dermal bone plates are made of thin, highly ordered sheets of mineralized bone matrix on the side facing the external environment on top of a thicker but less mineralized basal plate, which allows flexibility (De Vrieze *et al.*, 2012). In the fish integument, scales are found under the epidermis with its secreted mucus layer and on top of the dermis. Scales develop by intramembranous ossification, are remodelled extensively during growth and can keep growing peripherally by appositional growth (Ohira *et al.*, 2007; De Vrieze *et al.*, 2011). Peripheral cells and blood supply enable dynamic properties of scales, such as functions in calcium homeostasis (Mugiya and Watabe, 1977), sensitivity of mineralization to estrogens (Pinto *et al.*, 2017) and rapid regeneration abilities (Sire, 1989; Bereiter-Hahn and Zylberberg, 1993; Ohira *et al.*, 2007; De Vrieze *et al.*, 2011).

Because steroid hormones are known to accumulate in inert biological materials, we hypothesized that teleost fish scales can trap circulating steroids and release them at a much slower rate than short-term fluctuations in circulating plasma levels. This hypothesis postulates that teleost scales can reflect averaged steroid hormone levels through slow, passive diffusion exchange with neighbouring tissues and blood circulation. Estimates of averaged, chronic stress hormone levels in wild fish are of great interest but difficult to obtain due to the rapid elevation of stress hormones following capture (Pankhurst, 2011). To be useful as an indicator of chronic stress, scale cortisol content cannot be influenced by short-term fluctuations in the plasma after capture. Consequently, knowledge of the temporal profile of stress hormone accumulation and clearance in scales is crucial to interpret values in fish captured in their natural environment and to determine the methods of capture that are adequate for such studies. Here, we used goldfish as a model to study the accumulation and clearance of cortisol in scales under laboratory conditions. Specifically, we compared cortisol profiles in plasma and scales following a single acute physiological stressor, after hormone manipulation with cortisol implants, and in response to an unpredictable chronic stress (UCS) protocol. We predicted that each manipulation would produce slower increases followed by slower decreases in cortisol levels in scales compared to plasma. Additionally, because cortisol accumulation in the integument can differ spatially (Sharpley *et al.*, 2010), we investigated potential heterogeneity in scale cortisol content based on body location.

## Methods

### Fish

Mixed sex goldfish (*Carassius auratus*) of the common variety were obtained from AQuality (Mississauga, ON, Canada) and transported to the Hagen Aqualab at the University of Guelph (Guelph, ON, Canada). Fish were acclimated for at least 4 weeks in a common flow-through fiberglass tank (800 L) that was supplied with well water (pH 7.9; Ca^2+^, 2.7 mmol L^−1^; Cl^−^, 2.4 mmol L^−1^; Mg^2+^, 1.7 mmol L^−1^; K^+^, 0.05 mmol L^−1^; Na^+^, 1.9 mmol L^−1^), maintained at 20°C and exposed to a 12 h:12 h light:dark photoperiod regime. During both the acclimation and experimental phases of this study, all fish were fed *ad libitum* once daily with commercially prepared fish food (3PT Classic Sinking, Martin Mills, Elmira, Canada). All experimental protocols and procedures were approved by the University of Guelph local Animal Care Committee and were carried in accordance to the principles of the Canadian Council for Animal Care.

### Experimental design

#### Spatial heterogeneity of scale cortisol content

Six goldfish (119.1 ± 29.6 g, mean ± SEM) were removed from a common holding tank and terminally anaesthetized (5 mL L^−1^ 2-phenoxyethanol; Sigma-Aldrich) within a minute. The fish were weighed, and scales were removed with fine tweezers from five distinct locations on both flanks of the fish (Fig. 2A): (i) rostral scales were collected from the first four columns of scales extending posteriorly from the operculum; (ii) dorsal scales were collected from the three rows of scales next to the dorsal fin; (iii) midline scales were collected from five rows of scales centred on the lateral line; (iv) ventral scales were collected from the first three rows of scales next to the pelvic and anal fins; and (v) caudal scales were collected from the first four columns of scales extending anteriorly from the caudal fin. Scale samples were stored at −20°C in 20-mL glass scintillation vials until analysis of scale cortisol content.

#### Effects of an acute stressor on plasma cortisol and scale cortisol content

Five groups of nine fish each (37.7 ± 3.1 g, mean ± SEM; *n* = 45) were acclimated for a 3-week period in 80-L flow-through tanks. After this acclimation period, four groups were exposed to an acute stressor. The control undisturbed group was sampled at 10 a.m. immediately prior to administration of the acute stressor. The acute stressor treatment involved netting and air exposure for 2 min of all fish in a given tank prior to release. Treated fish were sampled 0.5, 4, 24 and 72 h after the acute stressor. All sampled fish were terminally anaesthetized (5 mL L^−1^ 2-phenoxyethanol) within 1 min of tank removal. A blood sample was immediately taken via caudal puncture using a K_2_EDTA (0.5 M, pH 8.0)-treated syringe. Blood samples were centrifuged at 14000 g for 3 min, and the separated plasma was stored at −20°C for later analysis of plasma cortisol as per Madison et al. (2015). All scales were removed from both flanks of the fish using a scalpel and stored at −20°C in 20-mL glass scintillation vials until analysis of scale cortisol content.

#### Effects of a cortisol implant on plasma cortisol and scale cortisol content

Six groups of eight fish each (30.0 ± 1.1 g, mean ± SEM; *n* = 48) were acclimated for a 3-week period in 30-L flow-through glass aquaria. After this acclimation period, five groups received a 300-μg g^−1^ body weight (BW) cortisol implant as per Bernier *et al.* (1999) and one group was left undisturbed. The control undisturbed group was sampled at 10 a.m. immediately prior to the implantation procedure. The implanted fish were first anesthetized in a buffered (NaHCO_3_, 0.4 g L^−1^) solution of tricaine methanesulfonate (0.2 g L^−1^; MS-222; Syndel, Vancouver, BC, Canada) and weighed. Cortisol was mixed with melted (30°C) cocoa butter (30 mg mL^−1^) and injected intraperitoneally (10 μL g^−1^ BW) with a 250-μL 18-gauge microsyringe (Bernier *et al.*, 1999). Injected fish were placed on ice for 1 min to promote solidification of the implants prior to returning the fish to their original tanks. Treated fish were sampled 8 h, 24 h, 3 days, 9 days and 27 days after receiving the implants. As in the acute stressor experiment above, all sampled fish were terminally anaesthetized, and both blood and scale samples were taken and stored for later analysis of plasma cortisol and scale cortisol content, respectively.

#### Effects of unpredictable chronic stress on plasma cortisol and scale cortisol content

Seven groups of fish (18.1 ± 0.5 g, mean ± SEM; *n* = 14–15 per group; total *n* = 103) were acclimated for a 3-week period in 80-L flow-through tanks. After this acclimation period, six groups were exposed once daily at 10 a.m. with one of five stressors and one group was left undisturbed before sampling. Treated fish were sampled after 7, 14 or 21 days of UCS or received UCS for 21 days and were sampled after 7, 14 or 21 days of recovery. The daily stressors included (i) rapid transfer from 20 to 12°C for 30 min and return to 20°C (low temp), (ii) rapid removal of tank water down to a 4-cm deep layer for 5 min and rapid refilling (low water), (iii) stirring the tank water with a net for 5 min (chasing), (iv) confinement to a dip net in shallow water for 5 min (netting) and (v) air exposure in a dip net for 2 min (air exposure). These stressors did not cause any physical damage such as scale loss or fin damage. The order of the five different stressors in the six treated groups was obtained using a random number generator, and the sequence was repeated as necessary. Fish in the 7-, 14- and 21-day UCS treatments were not stressed on the day of sampling and were always exposed to low water confinement as their last stressor prior to being sampled the next day. As in the acute stressor experiment above, all sampled fish were terminally anaesthetized, and both blood and scale samples were taken and stored for later analysis of plasma cortisol and scale cortisol content, respectively. Fish were not fed on the day of sampling, and all fish were sampled between 11:00 and 13:00.

To determine whether the individual stressors used in the UCS protocol do elicit an increase in plasma cortisol, six groups of fish (15.1 ± 0.8 g, mean ± SEM; *n* = 6–7 per group; total *n* = 40) were acclimated as above. A control group was left undisturbed and sampled. The other five groups were exposed to one of the five stressors used in the UCS protocol and sampled 30 min after stress initiation. All sampled fish were terminally anaesthetized as in the acute stressor experiment, and blood samples were taken and stored for later analysis of plasma cortisol. Fish were not fed on the day of sampling, and all fish were sampled between 11:00 and 13:00.

### Analytical techniques

#### Scale cortisol extraction

To remove surface mucus, scales were vortex-washed for 1 min twice using 10 mL of ultrapure water per wash. Scales were then transferred to a new 20-mL glass vial, dried to constant weight at 50°C and stored at −20°C until steroid extraction. From the dried scale sample, 50 mg was weighed into a new 20-mL glass vial to which 5 mL of methanol was added as extraction solvent.

To determine optimal methanol extraction duration, 50 mg of washed and dried scale samples (*n* = 5) were added to methanol and incubated for 72 h. The extraction solvent was separated from scales and replaced with 5 mL of fresh methanol at the 1-, 2-, 4-, 6-, 8-, 12-, 24- and 48-h incubation time points. Recovered supernatants were evaporated to dryness overnight under a stream of nitrogen gas, reconstituted with 1 mL of acetate buffer (2.35 mL glacial acetic acid, 0.74 g sodium acetate anhydrous, in 1 L; pH 4.0) and passed through C_18_ solid phase extraction (SPE) columns (Cleanert C18-N-SPE 100 mg mL^−1^; Agela Technologies, Wilmington, DE, USA) previously primed with 1 mL of methanol and 1 mL of ultrapure water. After the addition of samples to the C_18_ columns, 1 mL of ultrapure water and 1 mL of hexane were separately added and the eluates were discarded. Steroids were eluted from the SPE columns with 2 mL of ethyl acetate (containing 1% methanol) and collected in glass tubes. Samples were finally dried under nitrogen at room temperature, reconstituted in 700 μL of radioimmunoassay (RIA) buffer (21.4 mM Na_2_HPO_4_·7 H_2_O; 9.3 mM NaH_2_PO_4_·H2O; pH 7.6; 0.1% gelatin; 0.01% thimerosal) and stored at −20°C until cortisol measurement.

**Figure 1:**
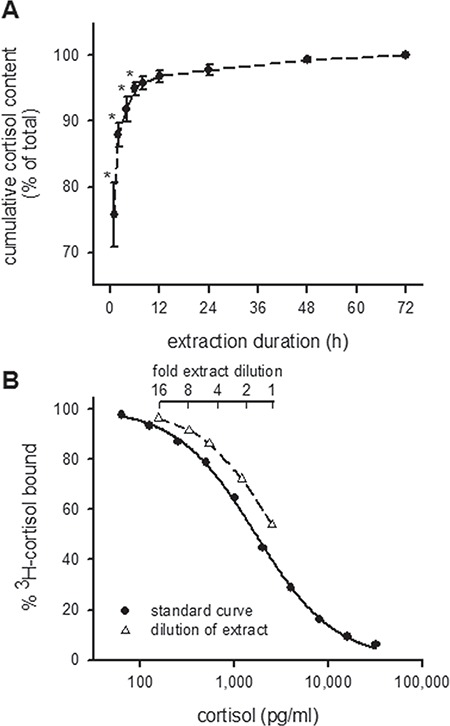
Extraction of cortisol from goldfish scales, and parallelism between diluted scale extract and RIA standards. (A) Relationship between extraction duration in methanol and percentage of total cortisol extracted from 50-mg scale samples over a 72-h incubation period (*n* = 5). Cumulative cortisol content values significantly lower than the 72-h extraction duration are indicated by asterisks. Data are mean ± SEM. (B) The displaced dilution curve of goldfish scale extract shows parallelism with the standard curve of the cortisol RIA

This pilot study demonstrated that the relationship between cumulative methanol cortisol content and incubation duration is hyperbolic (Fig. 1A). Relative to the 72-h cumulative methanol cortisol content, 75.7, 91.8 and 99.3% of scale cortisol was released into the extraction solvent after 1, 4 and 48 h of incubation, respectively. While cumulative methanol cortisol content after 1, 2, 4 and 6 h was lower than the 72-h value, the 8-, 12-, 24- and 48-h values were not. These results are supported by a significant Friedman test on ranks (*χ*^2^_8_ = 40.0, *P* < 0.001) and Dunn’s multiple comparison tests (*P* < 0.001). To estimate the recovery of hormone through the SPE columns, ^3^H-cortisol was added to scale samples during the addition of methanol and traced through the extraction process. The reconstituted extract contained 85.4 ± 2.2% (*n* = 4) of the ^3^H label.

In a separate pilot study, we compared methanol extraction of cortisol from minced and intact scales. In brief, washed and dried scales were either minced using a Euro Turrax T20b mechanical homogenizer (IKA Labortechnik, Staufen, Germany) or left intact. Minced or intact scale samples (50 mg) were then added to methanol and incubated for 72 h (*n* = 7 per treatment). Recovered supernatants were further purified using SPE columns as above and used to quantify cortisol content by RIA. Scale cortisol content did not differ between minced scales (6.73 ± 0.82 ng/g) and intact scales (7.02 ± 1.50 ng/g) as determined by Student’s *t* test (*P* = 0.87).

Based on the results from the above pilot studies, for each experimental sample, whole scales were placed on an orbital platform shaker at 100 rpm for 48 h at room temperature for cortisol extraction. The recovered supernatant fractions were processed as described above before cortisol measurement.

#### Cortisol measurement

Plasma cortisol and whole scale cortisol content were measured in triplicate 200-μL aliquots using a previously validated RIA (Bernier *et al.*, 2008). The anti-cortisol mouse monoclonal antibody used for this assay (product code XM210; Abcam, Cambridge, UK) has a high specificity for cortisol (*K*a = 1.7 × 10^9^ M^−1^) and is reported by the manufacturer to cross-react with 11-deoxycortisol (0.9%), corticosterone (0.6%), cortisone (0.6%), 11-deoxycorticosterone (<0.1%), progesterone (<0.1%), 17-hydroxyprogesterone (<0.1%), testosterone (<0.1%) and estradiol (<0.1%). The antibody was used at a final dilution of 1:5000. A serial dilution of scale extract gave a displacement curve that was parallel to the standard curve (Fig. 1B) indicating that the scale extract does not contain any substances that interfere with the cortisol RIA. The lower detection limits of the assay for plasma and scale samples were 62 pg mL^−1^ and 0.87 ng g^−1^ (2.39 pmol g^−1^), respectively. The intra- and interassay coefficients of variation for a commercial cortisol standard (product code 400364; Cayman Chemicals, Ann Arbor, MI, USA) diluted to 1000 pg mL^−1^ were 3.2% (*n* = 6) and 5.3% (*n* = 6), respectively. The intra- and interassay coefficients of variation for aliquots from a pool of scale extracts were 8.4% (*n* = 5) and 15.3% (*n* = 4), respectively. Scale cortisol content values are given as nanograms of cortisol per gram of dried scale and are corrected for the recovery of ^3^H-cortisol through the SPE columns.

### Statistics

Data for all experiments were analysed using one-way analysis of variance (ANOVA). Log_10_ transformation was applied if a Shapiro–Wilk test for normality or Bartlett test for homogeneity of variances among groups was significant. If log transformation proved insufficient to meet the ANOVA assumptions, the Kruskal–Wallis test on ranked data was used. Differences between controls and other groups were assessed using Dunnett’s or Dunn’s post-tests. In the spatial scale cortisol content experiment, comparisons between all body regions were done using the Tukey post-test. Linear regression was used to assess the relationships between body size, scale size and scale cortisol content. Data is presented as mean ± SEM, except when the Kruskal–Wallis test was used, where data is presented as median ± interquartile range. All tests were conducted in GraphPad Prism 7.04 (GraphPad Software, La Jolla, CA, USA) and *α* was set at 0.05.

## Results

### Spatial heterogeneity of scale cortisol content

**Figure 2:**
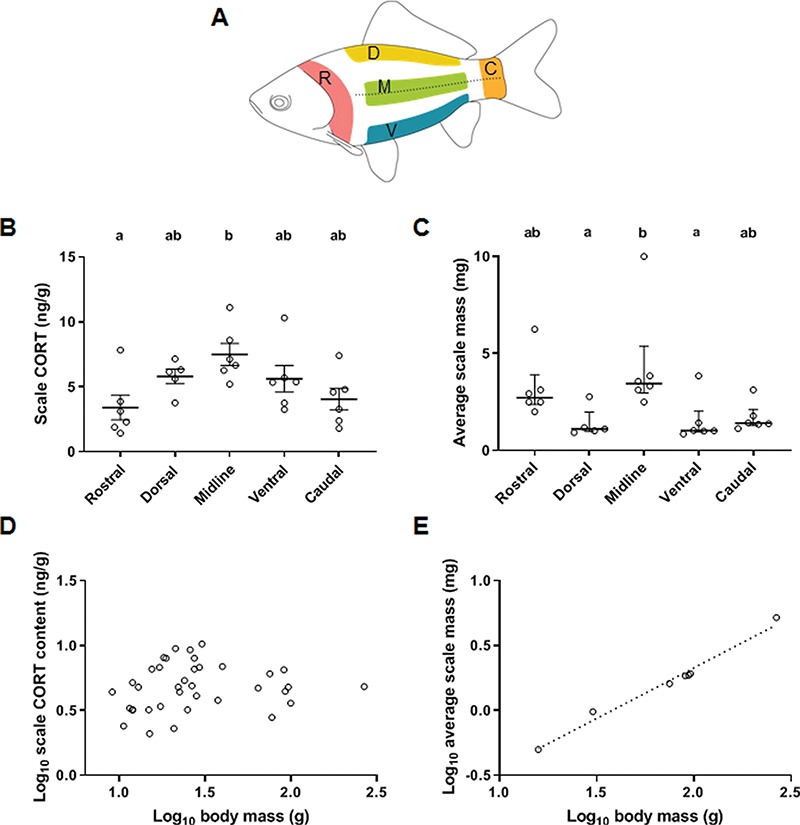
Spatial heterogeneity of scale cortisol (CORT) content. (A) Scales from five different body regions were analysed for cortisol content: R, rostral; D, dorsal; M, midline; V, ventral; C, caudal. (B) Regional cortisol content. Letters above regions show the output of Tukey’s post-test. Data are mean ± SEM. (C) Average scale mass across body regions. Letters above regions show the output of Dunn’s post-test on ranked data. Data are the median and interquartile range. Sample size is 6 per location, except for dorsal scales (*n* = 5). (D) Relationship between body mass and scale cortisol content. (E) Relationship between body mass and average scale mass

Scales from five different regions of the goldfish body were analysed for cortisol content (Fig. 2A). Overall, scale cortisol content was higher in midline scales compared to scales from rostral and caudal extremities (Fig. 2B). Specifically, the 2-fold higher cortisol levels in midline compared to rostral scales was statistically significant and the difference between midline and caudal scales approached statistical significance (ANOVA: *F*_4,24_ = 3.5, *P* = 0.02; Tukey’s: rostral vs. midline: *P* = 0.02; caudal vs. midline: *P* = 0.06; all other comparisons: *P* > 0.4). Even though these results should be taken with caution due to the small sample size (*n* = 5–6), they suggest that scales found across the body of fish accumulate cortisol to a different extent. Analysis of average scale mass across goldfish body regions showed that the regional difference in scale cortisol content is not due to a difference in scale size because midline scales were similar in mass to rostral scales, but heavier than scales in the dorsal and ventral body regions (Fig. 2C; Kruskal–Wallis: *H*_4_ = 15.1, *P* = 0.005; Dunn’s: dorsal vs. midline: *P* = 0.02; ventral vs. midline: *P* = 0.03; all other comparisons: *P* > 0.3). Another piece of evidence suggesting that scale cortisol content did not depend on scale size is the absence of relationship between body mass and scale cortisol content in control fish (Fig. 2D; *F*_1,35_ = 0.67, *P* = 0.42, *R*^2^ = 0.02) despite a positive relationship between body mass and scale mass (Fig. 2E; *F*_1,5_ = 247.5, *P* < 0.0001, *R*^2^ = 0.98).

### Effects of an acute stressor on plasma cortisol and scale cortisol content

**Figure 3:**
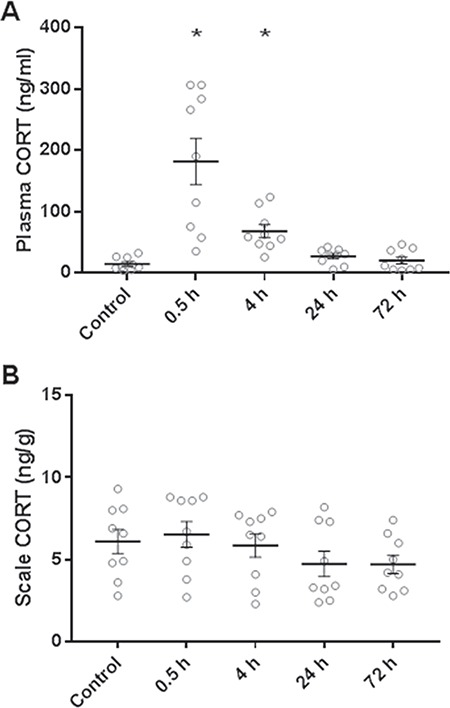
Effects of an acute stressor on plasma cortisol (CORT) and scale cortisol content over a period of 72 h. (A) Cortisol levels in plasma. (B) Cortisol levels in scales. Fish were exposed to air for 2 min before their return to their holding tank and sampling between 30 min and 72 h. Significantly increased levels compared to an unstressed control group are indicated by asterisks (*n* = 9). Data are mean ± SEM

Goldfish subjected to air exposure showed higher plasma cortisol levels 30 min (12-fold) and 4 h (4.6-fold) after the stressor, but plasma cortisol levels did not differ from control at later sampling times (Fig. 3A; ANOVA: *F*_4,40_ = 17.7, *P* < 0.0001; Dunnett’s: control vs. 0.5 h: *P* < 0.0001; 4 h: *P* = 0.0001; 24 h: *P* = 0.19; 72 h: *P* = 0.97). Despite this increase in the plasma, air exposure did not influence scale cortisol content (Fig. 3B; ANOVA: *F*_4,40_ = 1.34, *P* = 0.27).

### Effects of a cortisol implant on plasma cortisol and scale cortisol content

**Figure 4:**
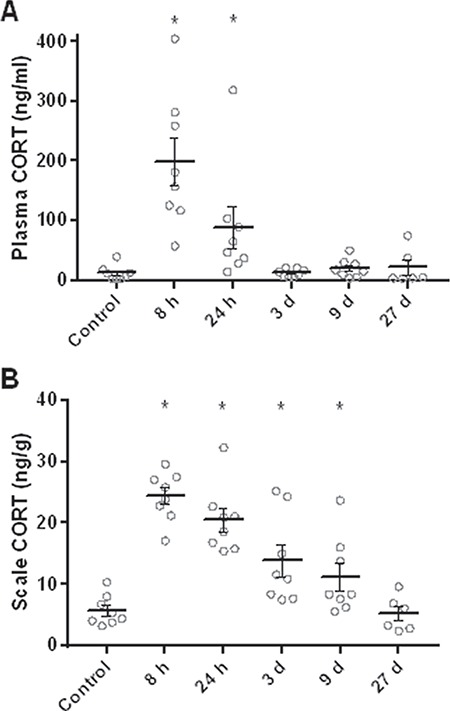
Effects of a cortisol (CORT) implant on plasma cortisol and scale cortisol content over a period of 27 days. (A) Cortisol levels in plasma. (B) Cortisol levels in scales. Significantly increased levels compared to an unstressed control group are indicated by asterisks. Sample size is 8 in all groups, except group 27 days (*n* = 6). Data are mean ± SEM

Goldfish treated with intraperitoneal implants of cortisol had mean plasma cortisol levels 16.3 times higher 8 h following injection and 7.2 times higher 24 h following injection, but cortisol levels had returned to baseline after 3 days (Fig. 4A). These differences are supported by a significant ANOVA (*F*_5,40_ = 14.2, *P* < 0.0001) and multiple comparisons using Dunnett’s test (control vs. 8 h: *P* < 0.0001; 24 h: *P* = 0.0004; 3 days: *P* = 0.94; 9 days: *P* = 0.52; 27 days: *P* = 1) on the log_10_-transformed data. The same fish showed a more persistent increase in scale cortisol content (Fig. 4B). Mean scale cortisol content was 4.2 times higher 8 h following injection and then decreased to about two times higher than control after 9 days. Scale cortisol content had returned to baseline 27 days after injection. These differences are supported by a significant ANOVA (*F*_5,40_ = 19.9, *P* < 0.0001) and Dunnett’s test (control vs. 8 h: *P* < 0.0001; 24 h: *P* < 0.0001; 3 days: *P* = 0.0009; 9 days: *P* = 0.02; 27 days: *P* = 0.93) on the log_10_-transformed data. Thus, use of the high-dose cortisol implant showed rapid increases in both plasma and scale cortisol contents at the earliest sampling time of 8 h. However, the decrease in cortisol content in scales was much slower since plasma cortisol levels had returned to baseline after 3 days and scale cortisol content was still significantly elevated after 9 days. It remained to be seen if physiologically relevant chronic stressors could increase scale cortisol content at all and if plasma and scale cortisol contents would increase at the same rate in the presence of what we expected would be lower circulating cortisol levels than those produced by a high-dose implant.

### Effects of unpredictable chronic stress on plasma cortisol and scale cortisol content

**Figure 5:**
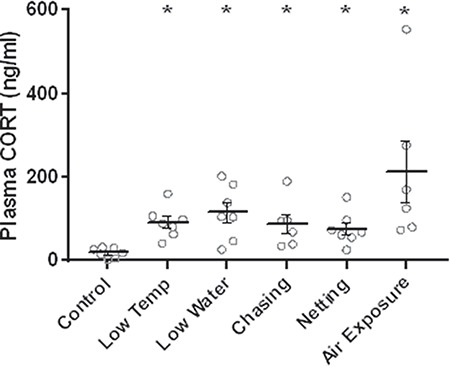
Validation of the five stressors used in the unpredictable chronic stress experiment. Significantly increased cortisol (CORT) levels compared to an unstressed control group are indicated by asterisks. Sample size is 7 per group, except for groups Chasing and Air Exposure (*n* = 6). Data are mean ± SEM

Before conducting the experiment using five different daily stressors, the effectiveness of each stressor in inducing an acute surge in cortisol in goldfish was verified. Figure 5 shows that each stressor was effective in increasing plasma cortisol levels 30 min after initiation of each stressor. An ANOVA and Dunnett’s test on the log_10_-transformed data confirmed that the observed increases were statistically significant (*F*_5,34_ = 9.5, *P* < 0.0001; all comparisons of control vs. stressor: *P* ≤ 0.001).

**Figure 6:**
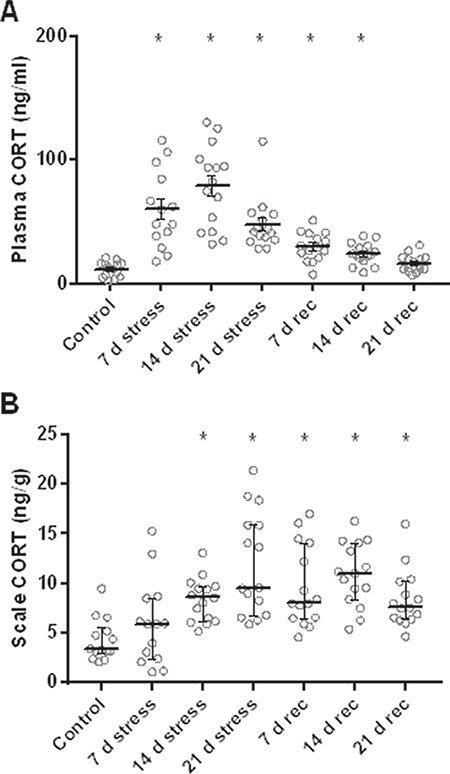
Effects of 21 days of unpredictable chronic daily stressors and 21 days of recovery from such stressors on plasma cortisol (CORT) and scale cortisol content. (A) Cortisol levels in plasma. (B) Cortisol levels in scales. Stressed fish were exposed daily to the stressors shown in Figure 5 in a pseudorandomized sequence for up to 21 days. Fish in recovery groups (rec) were exposed to 21 days of stressors before 1–3 weeks of recovery from stress. Significantly increased levels compared to an unstressed control group are indicated by asterisks. Sample size is 14 in control and 7-day groups and 15 in all other groups. Data are mean ± SEM in A and median and interquartile range in B

In the UCS experiment, daily administration of one of five different stressors increased plasma cortisol levels four to six times higher during the 21-day stress period (Fig. 6A). This increase was sustained at ~2.5 and 2 times higher than control after 7 and 14 days of recovery from 3 weeks of daily stressors, respectively, but plasma cortisol levels had returned to baseline after 21 days of recovery. These differences are supported by a significant ANOVA (*F*_6,96_ = 30.8, *P* < 0.0001) and multiple comparisons using Dunnett’s test (control vs. 7-day stress, 14-day stress, 21-day stress, 7-day rec and 14-day rec: all *P* < 0.0001; control vs. 21-day rec: *P* = 0.08) on the log_10_-transformed data. The UCS protocol also resulted in a delayed and more sustained increase in scale cortisol content compared to plasma (Fig. 6B). A detectable 2-fold increase in scale cortisol content was seen only after 14 days of daily stressors and scale cortisol levels remained two to three times higher than control for the rest of the experiment. These differences are supported by a significant Kruskal–Wallis test (*H*_6_ = 36.7, *P* < 0.0001) and Dunn’s multiple comparisons test (control vs. 7-day stress: *P* = 1; 14-day stress: *P* = 0.01; 21-day stress: *P* < 0.0001; 7-day rec: *P* = 0.003; 14-day rec: *P* < 0.0001; 21-day rec: *P* = 0.01). Thus, we observed that physiologically relevant stressors capable of producing a surge in circulating cortisol can increase scale cortisol content and that the rate of increase and decrease of this hormone is slower in scales compared to plasma.

## Discussion

For an indicator of chronic stress to be useful, measurements must meet three important criteria: (i) show no influence of the acute stress of capture, (ii) echo adrenal or interrenal activity and (iii) reflect the integrated stress hormone status over an extended period. Our results meet the above criteria. Namely, our results show that scale cortisol content (i) is not influenced by an acute emersion stressor, (ii) increases in response to a hypercorticoid state and chronic stress and (iii) increases more slowly and remains elevated longer than the changes in plasma cortisol. Overall, our novel data on the rates of scale cortisol accumulation and clearance in response to diverse stressors are consistent with the attributes of a chronic stress indicator and suggest that scales can provide an integrated measure of interrenal cell corticosteroid secretion activity. Moreover, our data demonstrate that scale cortisol content is spatially heterogeneous across the body surface of goldfish, identify potential limitations in the current use of scales as a minimally invasive indicator and provide perspective on the sensitivity of this matrix to echo interrenal activity.

We found that scale cortisol content varies among different body regions in goldfish independent of scale size. Teleost scales vary in morphology, composition and developmental rate depending on body location (Sire *et al.*, 1997; Ibañez *et al.*, 2009; Murcia *et al.*, 2017), which may contribute to heterogeneous cortisol accumulation. However, because we observed that scales from the midline region have higher cortisol content than in other regions, we suggest that increased vascularization along the midline region increases scale cortisol deposition. Scales in the midline region are located directly above and adjacent to the lateral line system, a structure that runs parallel to the lateral collecting vessel and that is supplied by a rich network of vessels from the secondary vascular system (Skov and Bennett, 2003; Bielecki *et al.*, 2008). In addition, located just beneath the integument of the midline region are distinct blocks of highly vascularized red muscle (Alexander, 1969). Therefore, although specific studies are needed to test the relationship between integument vascularization and scale cortisol content, we suggest that increased vascularization along the midline region leads to an increase in cortisol deposition into nearby scales due to proximity with blood circulation. An additional factor previously shown to affect glucocorticoid deposition in inert matrices is pigmentation. In birds, feather corticosterone levels are positively correlated with the amount of carotenoids in the feathers (Kennedy *et al.*, 2013; Lendvai *et al.*, 2013; Fairhurst *et al.*, 2014). Similarly, melanin concentration and type affect hair cortisol levels in some mammals (Bennett and Hayssen, 2010), but not in humans (Raul *et al.*, 2004; Sauvé *et al.*, 2007). While the relationship between epidermal pigmentation and cortisol content in scales remains to be determined, the highly variable interindividual colour pattern of goldfish suggests that local pigmentation does not explain the spatial heterogeneity in scale cortisol content observed here. Overall, as is done for mammalian hair (Sauvé *et al.*, 2007; Dettenborn *et al.*, 2012; Meyer *et al.*, 2014) and bird feathers (Romero and Fairhurst, 2016), we recommend that sampling procedures be standardized and take into account the spatial heterogeneity of scale cortisol content.

Results from the acute air exposure experiment suggest that scale cortisol content is not an indicator of acute stress. Aerial emersion is routinely used as a standardized acute stressor in fish (Barton and Iwama, 1991; Barton, 2002). In this study, air exposure was associated with a rapid but transient increase in plasma cortisol that recovered to baseline levels within 24 h. The absence of any change in scale cortisol content following this acute stressor suggest that accumulation in this matrix is not sensitive enough to detect short-lived physiological alterations in plasma cortisol levels. Similarly, short-term increases in plasma glucocorticoids following acute stressors do not affect hair cortisol (Ashley *et al.*, 2011; Grass *et al.*, 2015) and feather corticosterone (Fairhurst *et al.*, 2013; Romero and Fairhurst, 2016) levels. The apparent immunity of scale cortisol content from short-term fluctuations in plasma cortisol levels is an important finding towards the establishment of scale cortisol as an indicator of chronic stress. However, the standard field techniques used to capture (e.g. angling, netting, electrofishing) and hold fish post-capture prior to sampling may elicit greater and more sustained stress responses than the acute stressor used in this study (Pankhurst, 2011; Cooke *et al.*, 2013). Therefore, future studies are needed to determine whether scale cortisol content increases in response to a range of field capture techniques, and for those techniques that elevate scale cortisol content, efforts are needed to identify the minimum time interval between capture and scale cortisol content accumulation.

In contrast to the acute air exposure treatment, the cortisol implant treatment elicited a marked increase in scale cortisol content and a much slower clearance profile than the plasma cortisol response. The differential impact of the air exposure and cortisol implant treatments on scale cortisol content is consistent with the effect of these two treatments on plasma cortisol levels. Whereas plasma cortisol had returned to baseline levels within 24 h after the air exposure stressor, they were 7.2-fold higher than baseline 24 h post-injection in the cortisol implant treatment. Therefore, based on the results from a previous study in goldfish also using a 300-μg/g BW cortisol implant (Bernier *et al.*, 1999), and from a detailed evaluation of the steroid release profile from cocoa butter implants in goldfish (Pankhurst *et al.*, 1986), we conclude that the goldfish in the cortisol implant treatment were exposed to supraphysiological levels of cortisol. Nevertheless, consistent with Aerts *et al.* (2015) who reported a significant increase in scale cortisol content of common carp (*Cyprinus carpio*) fed a cortisol-spiked diet for 21 days, the result of our cortisol implant treatment show that scale cortisol content can be changed by a hypercorticoid state. Moreover, results from this cortisol manipulation treatment provide original information about the rate of cortisol clearance from scales. Following cortisol treatment, while plasma cortisol levels recovered to baseline levels within 3 days, scale cortisol content did not return to control levels until 27 days. Therefore, while scale cortisol content responds dynamically to changes in plasma cortisol over time, the rate of cortisol clearance from scales appears to be significantly slower than in plasma.

Besides inducing chronic stress and elevating scale cortisol content, the UCS experiment demonstrated that fish scales trap circulating cortisol and release it at a slower rate than plasma fluctuations. Although not stressed on sampling days, goldfish exposed to UCS had sustained increases in plasma cortisol throughout the 21-day protocol and for at least 14 days after cessation of the daily stressor administration. The effectiveness of our UCS protocol at elevating baseline cortisol levels over a period of several weeks is consistent with previous UCS experiments in zebrafish (Piato *et al.*, 2011; Manuel *et al.*, 2014; Song *et al.*, 2018) and suggests not only that the goldfish in this study did not habituate to the daily acute stressor but that the cumulative effect of the UCS protocol enhanced the activity of the neuroendocrine stress axis. In contrast, although the UCS protocol used by Aerts *et al.* (2015) induced an increase in the scale cortisol content of common carp, it did not elicit a sustained increase in plasma cortisol levels. Our results also show that in response to UCS, the increase in scale cortisol content lags at least 1 week behind the increase in plasma cortisol. However, we could not obtain a precise scale cortisol content clearance rate because scale cortisol values were still elevated above control levels after 21 days of recovery from the UCS protocol. Thus, it remains to be seen how long cortisol elevations attributable to the effects of chronic stressors persist in scales.

Potential limitations to the use of scale cortisol content as an indicator of chronic stress in fish include matrix sensitivity, assay sensitivity and assay specificity (see Methods for details). Overall, we observed that the magnitude of change in scale cortisol content in response to stressors was smaller than the fluctuations in plasma cortisol levels. Namely, the >16-fold supraphysiological increase in plasma cortisol levels following the cortisol implant treatment was matched with a 4.2-fold increase in scale cortisol content, and the 21-day UCS protocol was characterized by maximal 6.8- and 2.8-fold increases in plasma and scale cortisol levels, respectively. Therefore, while our laboratory experiments contribute to the validation of scale cortisol content as an indicator of chronic stress, it remains to be determined whether the sensitivity of this matrix will permit chronic stress monitoring in natural populations. Although Aerts *et al.* (2015) suggested that regenerated scales may be useful in providing better temporal resolution for scale cortisol incorporation, chronic daily stress for 21 days only increased cortisol content in ontogenetic but not regenerated scales in their study. In a subsequent study in milkfish, Hanke *et al.* (2019) showed that a 21-day exposure to constant high temperature increased cortisol accumulation in regenerated scales. Unfortunately, a comparison of cortisol accumulation in ontogenetic scales was not assessed after this exposure, preventing assessment of the benefit of using regenerated versus ontogenetic scales for cortisol measurements. Improvements in assay sensitivity will be needed before scale cortisol content can be considered a minimally invasive method for monitoring stress in small fish. Analysed by means of liquid chromatography tandem mass spectrometry (LC-MS/MS), Aerts *et al.* (2015) quantified scale cortisol content from 130 mg of sample which corresponded to five to six scales in ~360 g common carp. However, in Aerts *et al.* (2015), all control fish showed scale cortisol values that were below the detection capability of the LC-MS/MS protocol. Here, using a RIA, we quantified scale cortisol content from 50 mg of sample which corresponded to ~50 scales in 50 g fish or 10 scales in 270 g fish (see Fig. 2E for a relationship between body mass and average scale mass in goldfish). Finally, given that up to 10 different corticosteroids have been detected in human hair (Cirimele *et al.*, 2000), considerations must also be given to assay specificity. While the antibody used in this study has a high specificity for cortisol and a low cross-reactivity with structurally related compounds, both 11β-hydroxysteroid dehydrogenase type 2 (11β-HSD2) and 20β-HSD2, the enzymes involved in the conversion of cortisol to the inactive steroids cortisone and 20β-hydroxycortisone, respectively, are expressed in the skin of fish (Tokarz *et al.*, 2012). As such, further studies are required to investigate whether cortisone, 20β-hydroxycortisone and additional cortisol metabolites are deposited in fish scales and whether stressors affect the ratio of cortisol to its metabolites.

In conclusion, our results demonstrate that scale cortisol content can provide a time-integrated measure of interrenal cell cortisol secretion activity. However, unlike feather corticosterone levels which are frozen in time and only represent the period when the feather was actively growing (Romero and Fairhurst, 2016), or hair cortisol levels which provide a retrospective examination of cortisol deposition with hair growth (Russell *et al.*, 2012), our results suggest that cortisol exchange between the circulation and the scale matrix is dynamic. Therefore, fish scale cortisol content is unlikely to allow a retrospective historical assessment of stress status as far back in time as feather or hair corticosteroid content. The dynamic exchange of scale cortisol may only allow assessment of the most recent integrated cortisol status of fish. Overall, data from the cortisol implant and UCS experiments show that the rate of cortisol accumulation and clearance from scales depends on the magnitude of the difference in cortisol levels between the circulation and the scale matrix and that the lag needed to reach a new equilibrium between the two compartments after a hypercorticoid event may range between only hours to perhaps a few months. In contrast, the lack of change in scale cortisol content following a brief period of air exposure suggest that the contribution of any single acute stressor to scale cortisol content is likely quite small and that scales may not show short-lived fluctuations in plasma cortisol associated with capture stress. Therefore, although further studies are needed to determine the sensitivity of this matrix as an indicator of chronic stress, scale cortisol content may provide unique insights to understand whether long-term environmental perturbations are perceived as stressors, as well as the ability of fish to resist and recover from such perturbations.
